# Augmented risk of dementia in hypertrophic cardiomyopathy: A propensity score matching analysis using the nationwide cohort

**DOI:** 10.1371/journal.pone.0269911

**Published:** 2022-06-16

**Authors:** Heesun Lee, Hyung-Kwan Kim, Bongseong Kim, Kyungdo Han, Jun-Bean Park, In-Chang Hwang, Yeonyee E. Yoon, Hyo Eun Park, Su-Yeon Choi, Yong-Jin Kim, Goo-Yeong Cho

**Affiliations:** 1 Department of Internal Medicine, Seoul National University College of Medicine, Seoul, Korea; 2 Healthcare System Gangnam Center, Seoul National University Hospital, Seoul, Korea; 3 Cardiovascular Center, Seoul National University Hospital, Seoul, Korea; 4 Department of Statistics and Actuarial Science, Soongsil University, Seoul, Korea; 5 Department of Cardiology, Cardiovascular Center, Seoul National University Bundang Hospital, Seongnam, Gyeonggi, Korea; Karolinska Institutet, SWEDEN

## Abstract

**Background:**

Dementia is a big medical and socioeconomic problem on aging society, and cardiac diseases have already shown a significant contribution to developing dementia. However, the risk of dementia related to hypertrophic cardiomyopathy (HCM), the most common inherited cardiomyopathy, has never been evaluated.

**Methods:**

In a large-scale longitudinal cohort using National Health Insurance database, 4,645 subjects with HCM aged ≥50 years between 2010 and 2016 were collected and matched with 13,935 controls, based on propensity scores (1:3). We investigated the incidence and risk of dementia, Alzheimer’s disease (AD), and vascular dementia (VaD) between groups.

**Results:**

During follow-up (median 3.9 years after 1-year lag), incident dementia occurred in 739 subjects (4.0%): 78.2% for AD and 13.0% for VaD. The incidence of dementia, AD, and VaD were 23.0, 18.0, and 2.9/1,000 person-years, respectively, and was generally more prevalent in HCM. HCM group had a 50% increased risk of dementia, particularly AD, whereas there was no difference in the risk of VaD. The impact of HCM on AD (HR 1.52, 95% CI 1.26–1.84, *p*<0.001) was comparable with that of diabetes mellitus and smoking. Increased risk of AD in relation to HCM was consistent in various subgroups including younger healthier population.

**Conclusions:**

This is the first to demonstrate the increased risk of dementia, mainly AD rather than VaD, in subjects with HCM. Early surveillance and active prevention for cognitive impairment could help for a better quality of life in an era that HCM is considered a chronic manageable disease with low mortality.

## Introduction

Dementia is a neurodegenerative disease characterized by a progressive decline in brain function and global cognitive ability. Its prevalence is increasing owing to a demographic shift towards an elderly population [1, 2]. Global estimates suggest that 44 million people currently suffer from any type of dementia, and the number of patients are expected to quadruple by 2050, with the estimated total global societal cost of dementia exceeding $1.3 trillion [1, 3, 4]. As economic and public health challenges arising from dementia are increasing, clinical interest has focused on identifying risk factors for dementia and providing an appropriate preventive strategy to patients at high risk [5, 6].

Many previous studies demonstrated heart disease as one of the important risk factors for dementia [7–12]. Not only cardiovascular risk factors consistently showed a strong association with dementia [8, 9], but diverse cardiac diseases, including myocardial infarction (MI), atrial fibrillation (AF), and heart failure (HF), have also been reported to increase the risk of dementia [10–12]. Furthermore, dementia has recently been found to reflect the consequences of structural or functional cardiac abnormalities, such as left ventricular (LV) hypertrophy and diastolic dysfunction [13, 14]. Although hypertrophic cardiomyopathy (HCM), the most common inherited cardiomyopathy, is a cardiac disease representatively characterized by these two features, i.e. LV hypertrophy and diastolic dysfunction [15], no studies have evaluated the association between HCM and dementia. Currently, based on the advances in cardiac imaging technology and genetics, as well as evidence-based guidelines, accurate diagnosis, risk stratification, and effective management are achievable in major disease-related complications, including AF, stroke, HF, and sudden cardiac death [15, 16]. The majority of patients with HCM live their own lives, largely free from these complications, resulting in an annual mortality rate of <1% [16, 17]. Considering the increasing incidence and the extended lifespan of patients with HCM, it is timely to address the potential association between HCM and dementia and to deliberate the appropriate preventive strategy for cognitive dysfunction in HCM. Herein, we aimed to investigate the incidence and risk of dementia in subjects with HCM using a large-scale nationwide unselected cohort.

## Materials and methods

### Data source and study population

**Fig 1:**
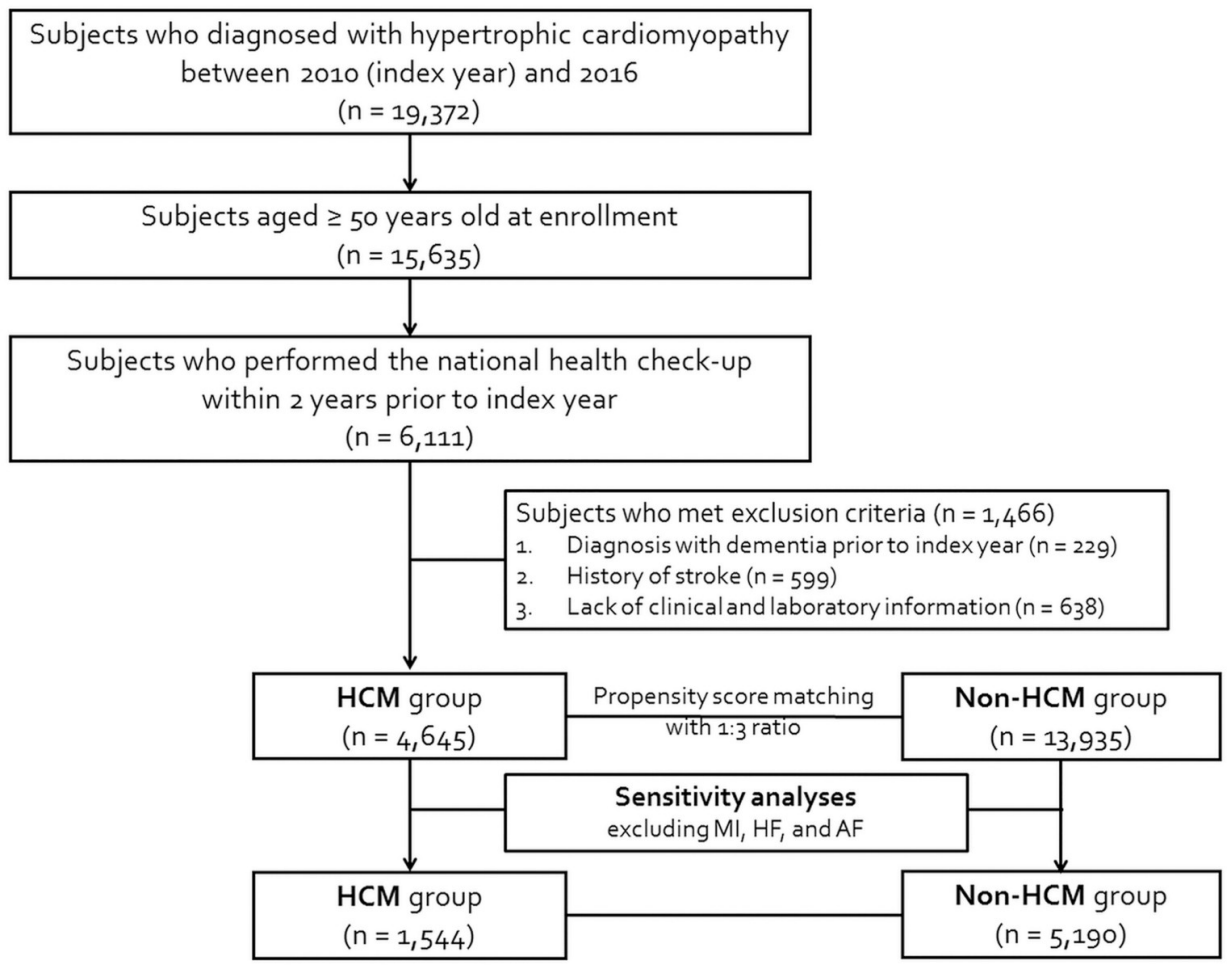
Schematic flow of the study population. AF, atrial fibrillation; HCM, hypertrophic cardiomyopathy; HF, heart failure; MI, myocardial infarction.

This nationwide population-based cohort study used data extracted from the National Health Insurance Service (NHIS) database. The NHIS is a mandatory universal health insurance program offering medical information of the entire Korean population, as described previously [18, 19]. The medical information by NHIS encompasses sociodemographic data, medical facility utilization history, diagnoses, prescriptions, treatments, and death information data. The Health Insurance Review and Assessment Service (HIRA) regularly reviews and validates the quality of the medical information under the strict supervision of the Ministry of Health and Welfare. In addition, all the insured adults aged ≥40 years are recommended to undergo a standardized national health examination biennially, which includes detailed surveys of demographics, medical histories and health-related behaviors, vital signs, anthropometric measurements, and laboratory tests. From this database, we collected individuals with HCM aged ≥50 years at enrollment and were diagnosed with HCM between January 1, 2010 and December 31, 2016 (n = 15,635). After excluding 9,524 subjects who did not undergo national health check-up within 2 years prior to enrollment, 229 previously diagnosed with any type of dementia, 599 with a prior history of stroke, and 638 with missing variables, a total of 4,645 subjects with HCM were finally included in the study. To balance the baseline clinical characteristics and to reduce selection bias and statistical inferences of confounders, we matched them with 13,935 non-HCM controls based on a propensity score (PS) for the final analysis (Fig 1).

The study protocol conformed to the ethical guidelines of the 1975 Declaration of Helsinki, and informed consent was waived since anonymized data were used retrospectively. This study was approved by the Institutional Review Board of Seoul National University Hospital (E-2001-057-1094).

### Definition of variables

HCM was defined as 1) at least one admission or outpatient clinic visit with the International Classification of Disease, 10^th^ Revision (ICD-10) codes (I42.1–42.2), and 2) registration in the Rare Intractable Diseases (RID) program (V127). Since 2006, the NHIS operates the RID program to provide special medical aid benefits to patients with diseases belonging to the program, such as HCM. As the Korean government covers 90% of all medical expenses claimed by these patients, the RID program is strictly monitored in accordance with an act established by the Ministry of Health and Welfare. For the RID registration of patients with HCM, attending physicians are obligated to fill out the application form with the following information: the ICD-10 code, diagnosis date, diagnostic methods, and the name and medical license number of the physicians who confirmed the final diagnosis. The HIRA then certifies the RID code of HCM by thorough verification for clinical and imaging evidence and periodic audit with external experts. Therefore, the definition of HCM used in this study is considered validated and reliable. Diagnosis of HCM based on the RID code was validated in the previous study of our institution by reviewing medical records including echocardiography or cardiac magnetic resonance imaging (MRI) of a random sample of 1,100 patients, demonstrating high diagnostic accuracy of 92.5% [20].

Definitions of other clinical and laboratory variables used in this study were the same as those previously published [18–21]. Age and sex data were retrieved from the resident registration number. Anthropometric measurements and blood pressures were collected by a trained nurse on the day of the assigned health exam. Health-related behaviors, including smoking status, alcohol consumption, and exercise were assessed using a self-reported questionnaire. Comorbidities were defined using the ICD-10 codes and/or the prescription lists from the NHIS database. Among the prescription lists, data on the renin-angiotensin-aldosterone system blockers, beta blockers, calcium-channel blockers, anti-platelet agents, and statins were separately obtained during the follow-up. All the laboratory results were reported from certified hospitals that were subjected to periodic quality control by the NHIS. They included hemoglobin, fasting glucose, serum total cholesterol, triglycerides, high-density lipoprotein cholesterol, low-density lipoprotein cholesterol, and estimated glomerular filtration rate level from the health exam results.

### Study endpoint and follow-up

The study population was followed up to the diagnostic date of dementia or until December 31, 2019, whichever came first. Subjects who died during follow-up or who did not develop dementia until the end of the study were censored. The primary endpoint was incident dementia during follow-up. The primary endpoint was collected from one year after the enrollment to minimize the reverse causality bias and elucidate the association. Incident dementia was defined based on ICD-10 codes (F00, F01, F02, F03, G30, or G31) along with the prescription of anti-dementia medications (acetylcholinesterase inhibitors or N-methyl-D-aspartate antagonists) for medical expense claims requested to the NHIS. The secondary endpoint was the development of Alzheimer’s disease (AD) (F00 or G30) or vascular dementia (VaD) (F01). If two or more dementia diagnosis codes were registered at the first visit, we deferred the decision for the type until the next visit. If multiple dementia codes were sustained at the next visit, we determined the dementia type based on the primary diagnosis. If all the dementia diagnosis codes were consistently registered as secondary diagnoses only, the case was determined as “other dementia” [22, 23].

### Statistical analysis

PS matching analysis with a 1:3 greedy technique was used to evaluate the independent association of HCM with dementia by reducing the potential selection bias due to confounding variables that might affect the risk of dementia. For calculating the PS, HCM (exposure) was determined as the dependent variable, and it was estimated by logistic regression using all variables in the baseline characteristics (shown in Table 1), including the known risk factors for dementia, as independent variables. Descriptive statistics are presented as mean ± standard deviation or median (interquartile ranges) for continuous variables and numbers (percentages) for categorical variables. The unpaired Student’s *t-*test for continuous variables and the χ^2^ test or Fisher’s exact test for categorical variables were used, as appropriate, to compare between the groups. The incidence rates of any type of dementia, AD, or VaD were calculated by dividing the number of detected cases by follow-up duration and were presented as a value per 1,000 person-years in the total cohort and the stratified subgroups. The confidence interval of the difference in the incidence rate between the groups was compared using χ^2^ test. The incidence probability was displayed between the groups by the Kaplan-Meier method with the log-rank test. The risk of dementia was assessed using Cox proportional hazards regression models and expressed as hazard ratios (HR) and corresponding 95% confidence intervals (CI) in the main analysis. Using the Schoenfeld residuals plot and log-log survival plot, the proportional hazards assumption was visually assessed. Stratified analyses were performed according to age, obesity, smoking, drinking status, income level, or comorbidities to evaluate the interactions between the subgroups, and the HRs of each subgroup were estimated using the interaction model. Furthermore, sensitivity analyses were performed exclusively in the subjects without serious medical conditions that could affect the development of dementia. Two-sided *p* values <0.05 were considered statistically significant. Statistical analyses were conducted using SAS version 9.4 (SAS Institute, Cary, NC, USA).

**Table 1:** Baseline characteristics of the study population according to HCM.

	Total (n = 18,580)	HCM (n = 4,645)	Control (n = 13,935)		ASD
***Demographics***					
Age, years	64.6 ± 8.9	64.5 ± 9.0		64.7 ± 8.8	0.022
50–59	6,070 (32.7)	1,649 (35.5)		4,421 (31.7)	0.080
60–69	6,429 (34.6)	1,559 (33.6)		4,870 (35.0)	0.029
≥70	6,081 (32.7)	1,437 (30.9)		4,644 (33.3)	0.051
Male sex	11,812 (63.6)	2,976 (64.1)		8,836 (63.4)	0.014
BMI, kg/m^2^	24.9 ± 3.1	24.9 ± 3.0		24.9 ± 3.2	0.001
BMI ≥25 kg/m^2^	8,842 (47.6)	2264 (48.7)		6578 (47.2)	0.031
Smoking					
Never	10,331 (55.6)	2,567 (55.3)		7,764 (55.7)	0.009
Ex	4,574 (24.6)	1,155 (24.9)		3,419 (24.5)	0.008
Current	3,675 (19.8)	923 (19.9)		2,752 (19.8)	0.003
Drinking					
No	11,156 (60.0)	2,763 (59.5)		8,393 (60.2)	0.015
Mild to moderate	6,097 (32.8)	1,546 (33.3)		4,551 (32.7)	0.013
Heavy	1,327 (7.1)	336 (7.2)		991 (7.1)	0.005
Systolic BP, mmHg	128.0 ± 15.5	127.9 ± 16.3		128.0 ± 15.2	0.003
Diastolic BP, mmHg	77.8 ± 10.1	77.4 ± 10.7		77.9 ± 9.9	0.054
Income lower 20%	3,032 (16.3)	754 (16.2)		2,278 (16.4)	0.003
***Previous medical history***					
Hypertension	10,713 (57.7)	2,631 (56.6)	8,082 (58.0)		0.027
Diabetes mellitus	3,743 (20.2)	909 (19.6)	2,834 (20.3)		0.019
Hypercholesterolemia	8,964 (48.3)	2,183 (47.0)	6,781 (48.7)		0.033
Myocardial infarction	771 (4.2)	189 (4.1)	582 (4.2)		0.005
Heart failure	2,659 (14.3)	687 (14.8)	1,972 (14.2)		0.018
Atrial fibrillation	1,629 (8.8)	460 (9.9)	1,169 (8.4)		0.053
***Medications***					
RAS blocker	9,662 (52.0)	2,356 (50.7)	7,306 (52.4)		0.033
CCB	4,973 (26.8)	1,187 (25.6)	3,786 (27.2)		0.037
BB	8,950 (48.2)	2,194 (47.2)	6,756 (48.5)		0.024
Anti-platelet agent	8,667 (46.7)	2,236 (48.1)	6,431 (46.2)		0.040
Anti-coagulant	791 (4.3)	234 (5.0)	557 (4.0)		0.050
Statin	7,902 (42.53)	1,927 (41.5)	5,975 (42.9)		0.028
***Laboratory findings***					
Hb, g/dL	14.2 ± 1.6	14.3 ± 1.6	14.2 ± 1.6		0.024
Total cholesterol, mg/dL	190.3 ± 39.5	190.8 ± 38.7	190.1 ± 39.8		0.019
HDL-cholesterol, mg/dL	51.6 ± 15.9	51.6 ± 15.3	51.5 ± 16.1		0.005
LDL-cholesterol, mg/dL	111.7 ± 43.4	112.2 ± 39.1	111.6 ± 44.7		0.014
Triglycerides, mg/dL	121.9 (121.0–122.8)	121.3 (119.5–123.1)	122.1 (121.1–123.1)		0.013
Glucose, mg/dL	104.2 ± 25.8	104.0 ± 25.9	104.3 ± 25.8		0.012
eGFR, mL/min/1.73m^2^	81.0 ± 34.1	81.0 ± 45.8	81.0 ± 29.2		0.003

Values are mean ± standard deviation, median (interquartile range), or n (%). ASD, absolute standardized difference; BB, beta blocker; BMI, body mass index; BP, blood pressure; CCB, calcium channel blocker; eGFR, estimated glomerular filtration rate; Hb, hemoglobin; HDL, high-density lipoprotein; HCM, hypertrophic cardiomyopathy; LDL, low-density lipoprotein; RAS, renin-angiotensin-aldosterone system.

## Results

### Baseline characteristics of the study population

The present cohort consisted of 4,645 subjects with HCM and 13,935 well-balanced PS-matched controls (n = 18,580; mean age 64.6 years; male 63.6%) (all the absolute standardized differences <0.1). The age groups in their 50s, 60s, and ≥70s accounted for a similar proportion of the study population at 32.7%, 34.6%, and 32.7%, respectively. The mean body mass index was 24.9 ± 3.1 kg/m^2^, with approximately half of the study population (47.6%) being obese. Current smoking and heavy alcohol drinking, both of which are well-known risk factors for dementia, were reported in 19.8% and 7.1% of the study population, respectively. In terms of comorbidities, 57.7%, 20.2%, and 48.3% of the total study participants had hypertension, diabetes mellitus, and hypercholesterolemia, respectively. A history of cardiovascular disease, including MI, HF, and AF were observed in 4.2%, 14.3%, and 8.8% of the subjects, respectively. Anti-platelet agents and anti-coagulants were prescribed in 46.7% and 4.3% of the subjects, respectively. More detailed data on baseline characteristics before and after PS matching are summarized in S1 Table and Table 1.

### Incidence of dementia

**Fig 2:**
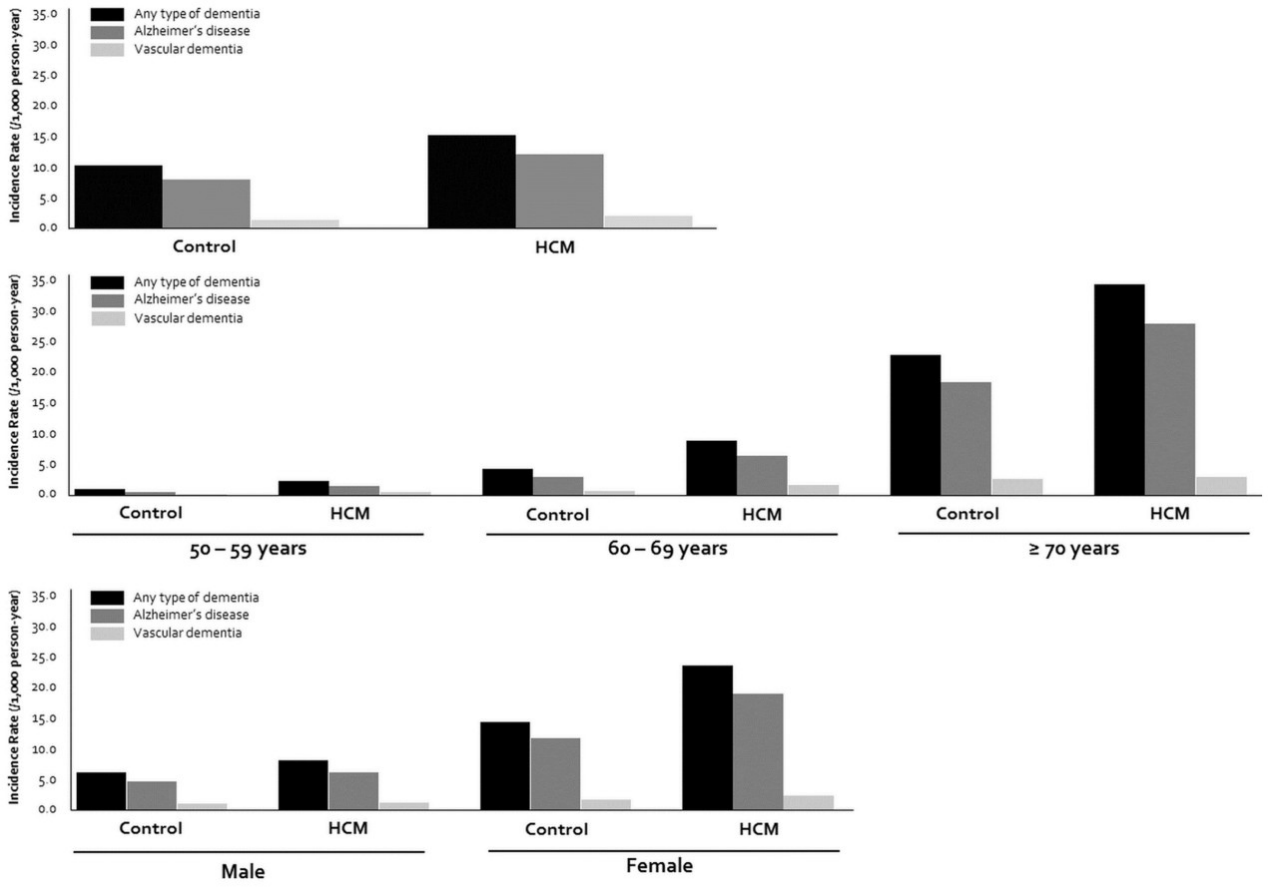
Comparison of incidence rates of any dementia, AD, and VaD according to age and sex. Generally, incidence rate of dementia was higher in patients with HCM than that in controls: AD was significantly frequent, whereas VaD did not have statistical significance. In age- and sex-stratified analyses, the incidence of dementia was higher in the elderly and women, regardless of dementia type. A consistent trend was found to have a higher incidence of any dementia and AD but a similar incidence of VaD in the HCM group. AD, Alzheimer’s disease, VaD, vascular disease, other abbreviation as Fig 1.

During a median follow-up of 3.9 years following a 1-year time lag, incident dementia was developed in 739 subjects (4.0%). Among them, 578 cases (78.2%) and 96 (13.0%) were attributable to AD and VaD, respectively. The incidence rates of any type of dementia, AD, and VaD were 23.0, 18.0, and 2.9 per 1,000 person-years, respectively. Subjects with any type of dementia were older, predominantly women, more likely to smoke and use excess alcohol, and had comorbidities, such as hypertension, diabetes mellitus, MI, HF, and AF, which were not different from the generally known characteristics of patients with dementia (S2 Table). When compared the incidence of dementia between subjects with HCM and controls, any type of dementia and AD were more prevalent in the HCM group (241 [5.2%] vs. 498 [3.6%] for any dementia, 189 [4.1%] vs. 389 [2.8%] for AD, all *p*<0.001). However, no significant difference was found in the incidence of VaD between the two groups (29 [0.6%] vs. 67 [0.5%], *p* = 0.154). In the age- and sex-stratified analyses, the incidence rates of any dementia and AD were also significantly higher in the HCM than in the control group, but that of VaD was not (Fig 2 and Table 2).

**Table 2:** Incidence and the risk of dementia in subjects with vs. without HCM.

	Any type of dementia				Alzheimer’s disease				Vascular dementia			
	Event (n)	IR*	HR (95% CI)	*p*	Event (n)	IR*	HR (95% CI)	*p*	Event (n)	IR*	HR (95% CI)	*p*
***Total population***												
Control	498	10.3	1.00 (ref.)	<0.001	389	8.0	1 (ref.)	<0.001	67	1.4	1 (ref.)	0.154
HCM	241	15.3	1.50 (1.27–1.78)		189	12.0	1.52 (1.26–1.84)		29	2.0	1.39 (0.88–2.22)	
***by Age group***												
***50–59***												
Control	18	1.0	1.00 (ref.)	<0.001	11	0.6	1.00 (ref.)	0.027	5	0.3	1.00 (ref.)	0.634
HCM	16	2.4	2.35 (1.20–4.61)		10	1.5	2.40 (1.02–5.64)		3	0.5	1.61 (0.38–6.72)	
***60–69***												
Control	82	4.3	1.00 (ref.)	<0.001	58	3.0	1.00 (ref.)	<0.001	16	0.8	1.00 (ref.)	0.081
HCM	53	8.9	2.11 (1.49–2.98)		39	6.5	2.20 (1.46–3.30)		11	1.8	2.23 (0.93–4.80)	
***≥70***												
Control	398	23.0	1.00 (ref.)	<0.001	320	18.5	1.00 (ref.)	<0.001	46	2.7	1.00 (ref.)	0.422
HCM	172	34.5	1.54 (1.29–1.84)		140	28.1	1.56 (1.28–1.91)		15	3.0	1.17 (0.65–2.10)	
***by Sex group***												
***Male***												
Control	213	6.2	1.00 (ref.)	0.003	156	4.6	1.00 (ref.)	0.010	33	1.0	1.00 (ref.)	0.344
HCM	93	8.2	1.33 (1.05–1.70)		70	6.2	1.37 (1.04–1.82)		14	1.2	1.29 (0.69–2.42)	
***Female***												
Control	285	14.4	1.00 (ref.)	<0.001	233	11.8	1.00 (ref.)	<0.001	34	1.7	1.00 (ref.)	0.225
HCM	148	23.7	1.67 (1.37–2.04)		119	19.1	1.65 (1.32–2.05)		15	2.4	1.43 (0.78–2.62)	

CI, confidence interval; HR, hazard ratio; IR, incidence rate; other abbreviations as Table 1.

### Risk factors for incident dementia

**Fig 3:**
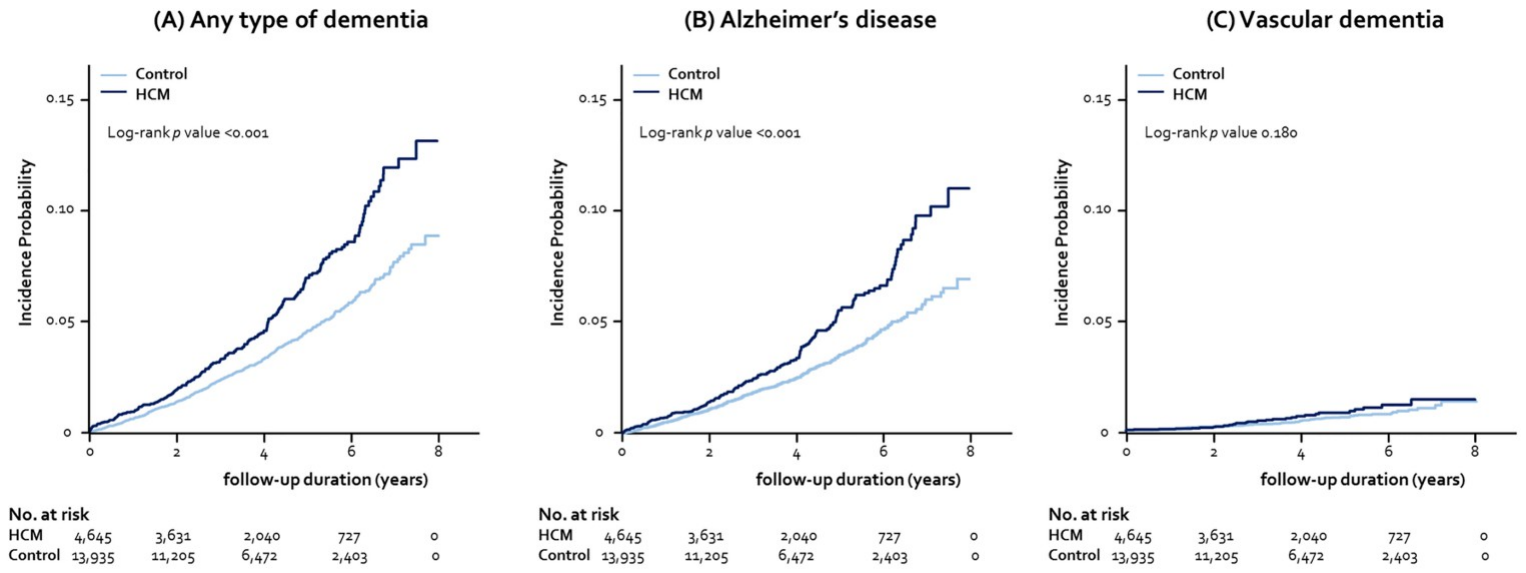
Kaplan-Meier curves for incidence probability of any dementia, AD, and VaD. The incidence probability of any type of dementia (A) and AD (B) tended to increase in patients with HCM, compared with controls. However, there was no significant differences in the incidence probability of VaD (C) the two groups. Abbreviations as Figs 1 and 2.

We explored the independent association between HCM and incident dementia in the PS-matched cohort (Table 2). The risk of any type of dementia was significantly higher by 50% in the HCM group, compared with the control group (HR 1.50, 95% CI 1.27–1.78, *p*<0.001). The close association of HCM with dementia seemed to be more predominant in AD, rather than in VaD. Subjects with HCM had a significantly increased risk of AD (HR 1.52, 95% CI 1.26–1.84, *p*<0.001), but not that of VaD (HR 1.39, 95% CI 0.88–2.22, *p* = 0.154), compared with controls. Kaplan-Meier curves also demonstrated higher incidence probabilities of any type of dementia and AD in subjects with HCM than in controls (log-rank *p*<0.001), but not VaD (log-rank *p* = 0.180) (Fig 3). Similar findings were noted in the age- and sex-stratified analyses, demonstrating the significant association of HCM with dementia, particularly AD, in all age and sex groups. Remarkably, HCM more than doubled the risk of developing any type of dementia, including AD, even in relatively young individuals in their 50s, although the absolute number of disease occurrence was small (HR 2.35, 95% CI 1.20–4.61, *p*<0.001 for any type of dementia; HR 2.40, 95% CI 1.02–5.64, *p* = 0.027 for AD). In addition, a consistent tendency was found in both men and women that HCM group had an increased risk of dementia and AD, but not of VaD (Table 2).

Cox regression analysis was performed to assess the impact of HCM on developing dementia, and to compare it with other known risk factors (Table 3). Age was the most powerful risk factor for any type of dementia, AD, and VaD, and more closely associated with AD than VaD. For men, the risk of dementia, particularly AD, was lower than for women. Smoking and heavy drinking were significantly associated with any type of dementia, AD, and VaD, whereas obesity had a protective effect on any type of dementia. Comorbidities, including hypertension, diabetes mellitus, MI, HF, and AF had a significant association with any type of dementia and AD. On the other hand, only hypertension, HF, and AF were independently associated with VaD. In this PS-matched cohort, the risk of dementia, particularly AD, was compatible with that of other known risk factors, such as diabetes mellitus (HR 1.49) and smoking (HR 1.57).

**Table 3:** The comparison of the impact on any type of dementia, Alzheimer’s disease, and vascular dementia by Cox regression analysis.

Variables	HR (95% CI)	*p*
**(A) Any type of dementia**		
HCM	1.50 (1.27–1.78)	<0.001
Age (per 10 years increment)	3.96 (3.60–4.36)	<0.001
Male sex	0.40 (0.35–0.47)	<0.001
Smoking	1.54 (1.45–1.61)	0.001
Heavy drinking	1.50 (1.27–1.66)	0.003
Obesity	0.68 (0.58–0.79)	<0.001
Income lower 20%	1.16 (0.96–1.40)	0.125
Hypertension	2.01 (1.74–2.54)	<0.001
Diabetes mellitus	1.40 (1.18–1.66)	<0.001
Hypercholesterolemia	1.06 (0.92–1.23)	0.411
Myocardial infarction	1.33 (1.15–1.53)	0.002
Heart failure	2.13 (1.81–2.52)	<0.001
Atrial fibrillation	1.99 (1.65–2.41)	<0.001
Prior use of statin	1.08 (0.94–1.25)	0.279
**(B) Alzheimer’s disease**		
HCM	1.52 (1.26–1.84)	<0.001
Age (per 10 years increment)	4.41 (3.95–4.92)	<0.001
Male sex	0.37 (0.31–0.43)	<0.001
Smoking	1.57 (1.48–1.64)	<0.001
Heavy drinking	1.70 (1.62–1.76)	<0.001
Obesity	0.70 (0.59–0.82)	<0.001
Income lower 20%	1.03 (0.83–1.29)	0.777
Hypertension	2.22 (1.78–2.76)	<0.001
Diabetes mellitus	1.49 (1.23–1.79)	<0.001
Hypercholesterolemia	1.10 (0.94–1.30)	0.235
Myocardial infarction	1.33 (1.12–1.56)	0.008
Heart failure	2.19 (1.82–2.64)	<0.001
Atrial fibrillation	1.81 (1.45–2.26)	<0.001
Prior use of statin	1.12 (0.95–1.32)	0.178
**(C) Vascular dementia**		
HCM	1.35 (0.87–2.08)	0.181
Age (per 10 years increment)	2.46 (1.93–3.14)	<0.001
Male sex	0.55 (0.37–0.82)	0.003
Smoking	1.43 (1.12–1.63)	0.011
Heavy drinking	1.36 (1.01–1.59)	0.045
Obesity	0.63 (0.42–0.95)	0.028
Income lower 20%	1.60 (1.00–2.58)	0.052
Hypertension	1.96 (1.17–3.27)	0.010
Diabetes mellitus	0.95 (0.56–1.60)	0.840
Hypercholesterolemia	1.02 (0.68–1.52)	0.931
Myocardial infarction	1.25 (0.83–1.87)	0.286
Heart failure	2.38 (1.52–3.71)	<0.001
Atrial fibrillation	2.93 (1.83–4.68)	<0.001
Prior use of statin	1.07 (0.71–1.60)	0.757

Abbreviations as Tables 1 and 2.

Furthermore, a sensitivity analysis was performed to verify the direct association between HCM and incident dementia, regardless of other cardiac diseases, such as MI, HF, and AF, previously proven to have a significant association with dementia, and similar results were obtained (S3 Table).

### Subgroup analysis

**Fig 4:**
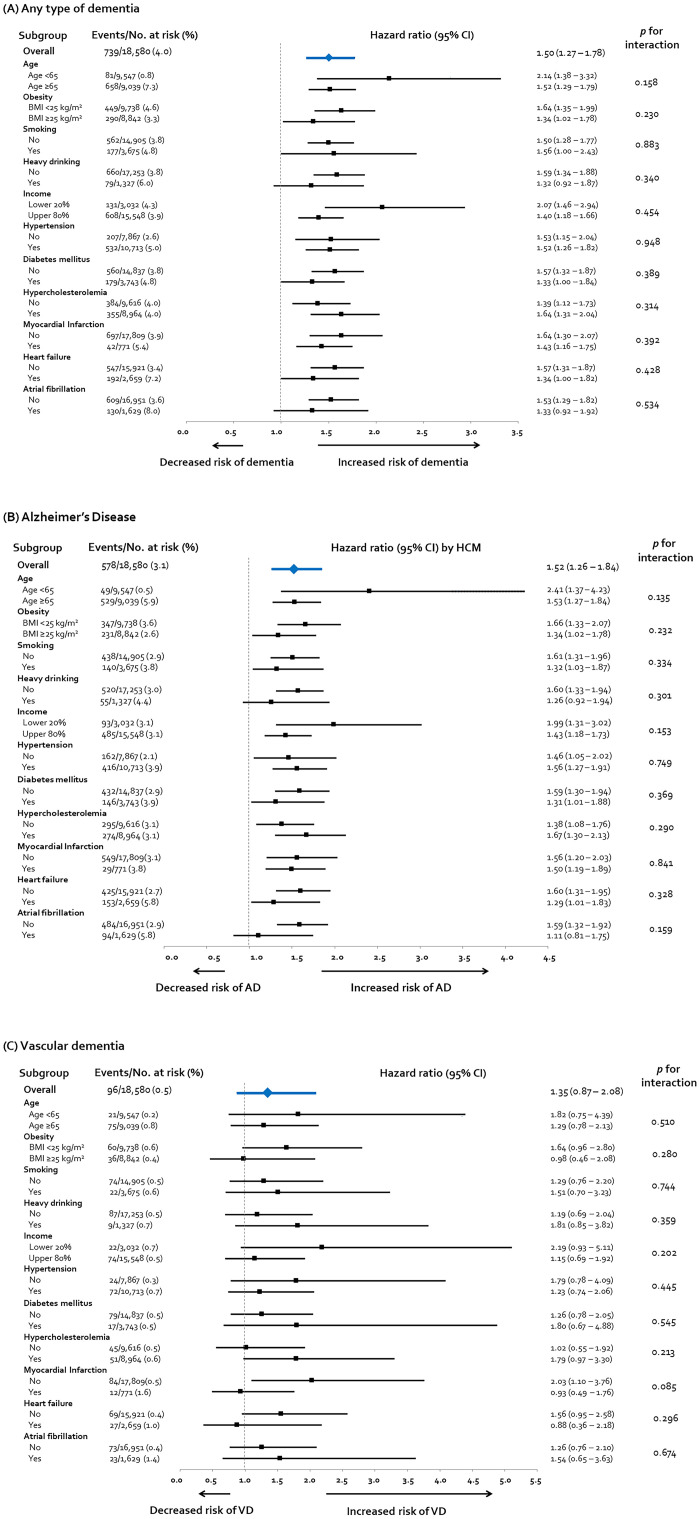
Subgroup analysis regarding the risk of AD by HCM. HCM showed a consistent tendency to increase the risk of any type of dementia (A) and AD (B), in almost all subgroups stratified by age, life behavior, and comorbidity. In particular, subgroups that were deemed low risk for dementia demonstrated a high risk for developing AD by HCM, suggesting the direct impact of HCM on cognitive dysfunction. On the other hand, the risk of VaD did not show a significant increase in all subgroups except for the subgroup without a history of MI. BMI, body mass index; CI, confidence interval; other abbreviations as Figs 1 and 2.

In line with the main analysis, HCM showed a consistent tendency to be associated with the increased risk of dementia, particularly AD, in almost all the subgroups stratified by age, life behavior, and comorbidities (Fig 4A and 4B). Despite no statistical significance of *p*-for-interaction value, the risk of dementia in the HCM group seemed to be attenuated in heavy drinkers and having AF, implying a strong impact of heavy drinking and AF itself on developing dementia, particularly AD. In the current analysis, the HRs of heavy drinking and AF for developing AD were slightly greater than that of HCM. Of note, HCM subgroups that were deemed low risk for cognitive impairment, i.e., <65 years old, non-obese, non-smoker, non- or social drinker, and no history of comorbidities, such as diabetes mellitus, MI, HF, and AF, demonstrated a high risk for developing AD. On the contrary, none of the HCM subgroups had an increased risk for VaD (Fig 4C).

## Discussion

The main findings of this large-scale nationwide PS-matched cohort study involving 18,580 subjects aged ≥50 years during a median follow-up of 3.9 years are summarized as follows. 1) The overall incidences of any type of dementia, AD, and VaD were 23.0, 18.0, and 2.9 per 1,000 person-years, respectively, and were generally more prevalent in subjects with HCM, compared with the PS-matched controls, 2) individuals with HCM had a 50% increased risk of incident dementia, particularly AD, but no significant difference was found in the risk of VaD, compared with controls, 3) the impact of HCM on developing AD was compatible with that of diabetes mellitus and smoking, and 4) the risk of AD was more evident, even in younger and healthier subjects with HCM. Particularly, all these findings are observed after ‘*1-year lag*’ of data collection after enrolling the study participants, to minimize the reverse causality bias. As far as we know, this study is the first to demonstrate the heightened risk of dementia, particularly AD, and introduces HCM as a novel risk factor for dementia, suggesting that early surveillance and active preventive strategy for cognitive impairment are required.

Various cardiac diseases have been suggested and proven as significant risk factors for dementia [7–12]. It is plausible that cerebral hypoperfusion could arise from hemodynamic disturbances caused by cardiac diseases. In addition, cardiac disease and dementia could potentially share a common pathophysiology–atherosclerosis, thromboembolism, and etc [9]. However, its relationship with HCM is not known to date, despite the increasing prevalence and clinical significance of the disease.

In this large-scale longitudinal study with a 1-year lag and approximately 4-year follow-up, we demonstrated that the risk of dementia significantly increased in subjects with HCM aged ≥50 years. Considering that the present study showed a similar prevalence of comorbidities, such as hypertension, diabetes mellitus, and hypercholesterolemia, to that in the general Korean population [24–26], the study population could be considered not skewed and the study results are potentially generalizable. We compared the risk of dementia in HCM group with that in the PS-matched controls, emphasizing an independent association of HCM with the increased risk of dementia. Additional analyses after excluding other cardiac diseases that previously showed a close relationship with dementia (sensitivity analysis) or stratifying the subjects according to demographics and comorbidities (subgroup analysis) also verified that HCM had an independent association with the increased risk of incident dementia, rather than merely coincided with dementia. Our results suggest the possibility of HCM as a new risk factor for dementia. Active surveillance and early intervention for preventing cognitive dysfunction need to be considered in subjects with HCM to potentially improve their quality of life.

One of the remarkable findings in the current study was that the augmented risk of dementia in subjects with HCM was mainly noted in AD, not in VaD. Until now, the main concern related to cognition in patients with HCM has been ischemic stroke and its potential sequelae, such as VaD. AF is the most common arrhythmia in HCM, and the current guidelines strongly recommend anticoagulation in all patients with HCM having a history of clinical AF, irrespective of CHA_2_DS_2_-VASc score [16]. This recently reinforced recommendation for anticoagulation therapy aimed at preventing thromboembolic events, including stroke and subsequent VaD, which basically originate from AF. Furthermore, recent studies have shown that patients with HCM even without a history of documented AF have a high probability of embolic events, including stroke, alluding that HCM itself could be considered a risk factor for ischemic stroke and subsequent VaD [27, 28]. However, given that the most affected patients with HCM in earlier studies were elderly with the enlarged left atrium (LA), they were presumed to be predisposed to the development of AF—an asymptomatic paroxysmal episode or a first paroxysmal episode of AF—rather than HCM itself [27]. In light of this, this issue needs to be re-evaluated in a large cohort, where the prevalence of AF and history of anticoagulation are preemptively balanced. After the PS-matching with multiple known risk factors, we observed that there was no difference in the incidence and the risk of VaD between the HCM group and controls. In contrast, the incidence and the risk of AD consistently increased in all HCM subgroups as well as in the total cohort. Taken together, the occurrence of AD was speculated to be related to HCM *per se*, and VaD seemed to be attributed to concomitant AF in subjects with HCM. Particularly, the association of HCM with AD was more prominent in the younger and healthier subjects, subgroups deemed at low risk of developing dementia. Thus, it is reasonable to periodically screen cognitive impairment in subjects with HCM to promptly detect dementia and AD and proactively manage its risks, apart from the anticoagulation therapy recommended in the current guidelines. However, the current cohort demonstrated a lower prevalence of VaD than previously published epidemiologic data [29], possibly due to relatively a short follow-up period and exclusion of subjects who had a prior history of stroke. Hence, caution should be taken in interpreting the results.

Since the present study did not interrogate the causality or mechanism, it should be acknowledged that the mechanisms underlying the association of HCM with dementia, particularly AD, are not certain. We assume that the cognitive dysfunction in HCM may be attributed mainly to LV diastolic dysfunction induced by hypertrophied LV. Theoretically, LV diastolic dysfunction by hypertrophied LV in HCM can decrease LV inflow from LA, and consequently reduce the LV stroke volume, causing systemic hypoperfusion. Accordingly, it can worsen the autoregulatory system of the cerebral blood flow, disrupt cerebral perfusion, and decrease cerebral arteriolar compliance, all of which can account for diffuse small vessel disease and cognitive impairment [30].

As cognitive impairment initially emerged as a problem in HF, prior studies have investigated the association between global LV systolic dysfunction and cognitive impairment; however, conflicting results were also reported [31, 32]. Thereafter, several studies have provided robust evidence that LV diastolic, not systolic, dysfunction, is closely associated with the development of dementia and AD [13, 32–35]. In the population-based Rotterdam study consisting of 3,291 individuals aged 58–98 years, measures of better LV diastolic function reduced the risk of stroke and dementia by 28% [32]. Similarly, Calik *et al*. demonstrated that deteriorated LV diastolic function was more prevalent in patients with AD, compared with controls [35]. Some subsequent studies have supported this finding using brain imaging [34, 36–38]; demonstrating that periventricular white matter hyper-intensities on the brain MRI, known to be related to the risk of AD [37], have a quantitative association with LV diastolic dysfunction in various ethnicities [36, 38]. In other studies, a strong association was noted between LV diastolic dysfunction and decline in the multiple domains of cognitive function, such as working memory, fluency, attention, and execution [33, 34]. Considering these results, LV diastolic dysfunction, one of the main pathophysiology in HCM, could make the structural and functional changes in the brain, and be linked to cognitive impairment and development of dementia, particularly AD. Although a question still remains as to why HCM is related to dementia, particularly AD, but not to VaD. Further studies are warranted to explore this issue.

There are several imitations in this study. First, we defined all the diseases, including HCM and dementia, based on the diagnostic codes of claims data. In terms of HCM, we could not access echocardiographic or cardiac MRI results of each subject, and thus, a piece of the study participants might be misdiagnosed with HCM. Moreover, HCM severity could not be analyzed. Likewise, since we did not possess all the pertinent information on dementia and its types, such as cognitive function tests, *APOE4* carrier status, and education or literacy levels, the accuracy of the study outcomes might be affected. However, in Korea, the final diagnosis of HCM and dementia depends on the clinical and/or imaging evidence strictly validated by health insurance professionals and external medical experts. In particular, subjects with HCM in this study were exclusively collected from the RID program registration, which has already been previously validated, presenting a high positive predictive value of ICD code-based HCM definition [20]. In addition, HIRA strictly reviews and supervises the medical claims of anti-dementia medications that were involved in our definition of dementia [39, 40]. That is, rigorous standards, including mini-mental state examination, clinical dementia rating, and global deterioration scale, should be met in order to consider prescribing the related medications under the insurance coverage by the Korea government [41]. Hence, the potential validity issues can be alleviated, and the diagnostic reliability of this study can be strengthened despite the use of the claims data. Second, we could not fully reveal the pathophysiology of how HCM was associated with dementia, particularly AD. Because of the observational nature of the study with a relatively short-term follow-up period, a causal relationship could not be assessed. Well-designed prospective studies are required to explore the causality and mechanism of HCM-related cognitive dysfunction. Finally, the role of unrecognized confounders that could affect cognition could not be completely excluded, even though we preemptively matched the established risk factors before the analysis. The obtained findings should be validated in the future.

## Conclusions

This large-scale nationwide PS-matched cohort study is the first to demonstrate the augmented risk of dementia, particularly AD, in subjects with HCM. Therefore, active surveillance and early preventive strategy for cognitive impairment could potentially improve the quality of life in HCM in an era where HCM is considered a chronic manageable disease with low mortality.

## Supporting information

S1 Table Baseline characteristics of the study population according to HCM before matching propensity score. (DOCX)

S2 Table Baseline characteristics of the study population according to incident dementia. (DOCX)

S3 Table The comparison of the impact on the dementia and its subtypes after excluding other cardiac diseases. (DOCX)

S1 Checklist STROBE statement—Checklist of items that should be included in reports of *cohort studies*. (DOCX)
