# Supplementary material for: Augmented risk of dementia in hypertrophic cardiomyopathy: A propensity score matching analysis using the nationwide cohort
Source: PLoS One. 2022 Jun 16;17(6):e0269911. doi: 10.1371/journal.pone.0269911 (PMC9202937; doi:10.1371/journal.pone.0269911)
Supplement: S1 Table — (DOCX) [file pone.0269911.s001.docx]

**Supplementary Table 1. Baseline characteristics of the study population according to HCM before matching propensity score.**

|  | Total  (n = 19,072) | HCM  (n = 4,768) | Control  (n = 14,304) | | *p* |
| --- | --- | --- | --- | --- | --- |
| ***Demographics*** | | | | | |
| Age, years | 64.6 ± 8.9 | 64.5 ± 9.0 | | 64.7 ± 8.8 | 0.999 |
| 50 – 59 | 7,316 (38.4) | 1,829 (38.4) | | 5,487 (38.4) |  |
| 60 – 69 | 6,772 (35.5) | 1,693 (35.5) | | 5,079 (35.5) |  |
| ≥70 | 4,984 (26.1) | 1,246 (26.1) | | 3,738 (26.1) |  |
| Male sex | 12,632 (66.2) | 3,158 (66.2) | | 9,474 (66.2) | 0.999 |
| BMI ≥25 kg/m^2^ | 7,796 (40.9) | 2,400 (50.3) | | 5,396 (37.7) | <0.001 |
| Smoking |  |  | |  | 0.016 |
| Never | 10,395 (54.5) | 2,513 (52.7) | | 7,882 (55.1) |  |
| Ex | 4,791 (25.1) | 1,243 (26.1) | | 3,548 (24.8) |  |
| Current | 3,886 (20.4) | 1,012 (21.2) | | 2,874 (20.1) |  |
| Drinking |  |  | |  | 0.449 |
| No | 11,067 (58.0) | 2,739 (57.5) | | 8,328 (58.2) |  |
| Mild to moderate | 6,649 (34.9) | 1,673 (35.1) | | 4,976 (34.8) |  |
| Heavy | 1,356 (7.1) | 356 (7.5) | | 1,000 (7.0) |  |
| Income lower 20% | 4,104 (21.5) | 783 (16.4) | | 3,321 (23.2) | <0.001 |
| ***Previous medical history*** | | | | | |
| Hypertension | 10,839 (56.8) | 3,306 (69.3) | 7,533 (52.7) | | <0.001 |
| Diabetes mellitus | 3,793 (19.9) | 904 (19.0) | 2,889 (20.2) | | 0.064 |
| Hypercholesterolemia | 7,151 (37.5) | 2,244 (47.1) | 4,907 (34.3) | | <0.001 |
| Myocardial infarction | 365 (1.9) | 187 (3.9) | 178 (1.2) | | <0.001 |
| Heart failure | 1,917 (10.1) | 889 (18.7) | 1,028 (7.2) | | <0.001 |
| Atrial fibrillation | 1,157 (6.1) | 586 (12.3) | 571 (4.0) | | <0.001 |
| ***Medications*** | | | | | |
| RAS blocker | 7,186 (37.7) | 2,389 (50.1) | 4,797 (33.5) | | <0.001 |
| CCB | 1,323 (6.9) | 865 (18.1) | 458 (3.2) | | <0.001 |
| BB | 4,953 (26.0) | 2,423 (50.8) | 2,530 (17.7) | | <0.001 |
| Anti-platelet agent | 5,538 (29.0) | 2,313 (48.5) | 3,225 (22.6) | | <0.001 |
| Anti-coagulant | 720 (3.8) | 319 (6.7) | 401 (2.8) | | <0.001 |
| ***Laboratory findings*** | | | | | |
| Hb, g/dL | 14.1 ± 1.6 | 14.4 ± 1.6 | 14.0 ± 1.6 | | <0.001 |
| Total cholesterol, mg/dL | 192.9 ± 39.0 | 190.8 ± 38.6 | 193.6 ± 39.1 | | <0.001 |
| HDL-cholesterol, mg/dL | 52.8 ± 17.0 | 51.5 ± 17.5 | 53.2 ± 16.8 | | <0.001 |
| LDL-cholesterol, mg/dL | 113.1 ± 37.2 | 112.1 ± 39.0 | 113.4 ± 36.6 | | 0.045 |
| Triglycerides, mg/dL | 120.0  (119.1-120.9) | 122.7  (120.9-124.5) | 119.15  (118.1-120.2) | | 0.001 |
| Glucose, mg/dL | 105.2 ± 28.6 | 103.7 ± 25.4 | 105.8 ± 29.6 | | <0.001 |

Values are mean ± standard deviation, median (interquartile range), or n (%). ASD, absolute standardized difference; BB, beta blocker; BMI, body mass index; BP, blood pressure; CCB, calcium channel blocker; eGFR, estimated glomerular filtration rate; Hb, hemoglobin; HDL, high-density lipoprotein; HCM, hypertrophic cardiomyopathy; LDL, low-density lipoprotein; RAS, renin-angiotensin-aldosterone system.
