# Supplementary material for: Augmented risk of dementia in hypertrophic cardiomyopathy: A propensity score matching analysis using the nationwide cohort
Source: PLoS One. 2022 Jun 16;17(6):e0269911. doi: 10.1371/journal.pone.0269911 (PMC9202937; doi:10.1371/journal.pone.0269911)
Supplement: S2 Table — (DOCX) [file pone.0269911.s002.docx]

**Supplementary Table 2. Baseline characteristics of the study population according to incident dementia**

|  | Total  (n = 18,580) | Incident dementia | | | | *p* |
| --- | --- | --- | --- | --- | --- | --- |
|  |  | Yes (n=739) | | | No (n=17,841) |  |
| ***Demographics*** | | | | | | |
| Age, years | 64.6 ± 8.9 | 73.7 ± 7.2 | | 64.3 ± 8.7 | | <0.001 |
| 50 – 59 | 6,070 (32.7) | 34 (4.6) | | 6,036 (33.8) | | <0.001 |
| 60 – 69 | 6,429 (34.6) | 135 (18.3) | | 6,294 (35.3) | |  |
| ≥70 | 6,081 (32.7) | 570 (77.1) | | 5,511 (30.9) | |  |
| Male sex | 11,812 (63.6) | 306 (41.4) | | 11,506 (64.5) | | <0.001 |
| BMI, kg/m^2^ | 24.9 ± 3.1 | 24.3 ± 3.2 | | 24.9 ± 3.1 | | <0.001 |
| BMI ≥25 kg/m^2^ | 8,842 (47.6) | 290 (39.2) | | 8,552 (47.9) | | <0.001 |
| Smoking | 3,675 (19.8) | 177 (24.0) | | 3,498 (19.6) | | 0.015 |
| Heavy drinking | 1,327 (7.1) | 79 (10.7) | | 1,248 (7.0) | | <0.001 |
| Systolic BP, mmHg | 128.0 ± 15.5 | 127.9 ± 15.4 | | 129.7 ± 16.3 | | 0.001 |
| Diastolic BP, mmHg | 77.8 ± 10.1 | 77.8 ± 10.1 | | 77.7 ± 10.9 | | 0.788 |
| Income lower 20% | 3,032 (16.3) | 131 (17.7) | | 2,278 (16.4) | | 0.291 |
| ***Previous medical history*** | | | | | | |
| Hypertension | 10,713 (57.7) | 532 (72.0) | 10,181 (57.1) | | | <0.001 |
| Diabetes mellitus | 3,743 (20.2) | 179 (24.2) | 3,564 (20.0) | | | 0.005 |
| Hypercholesterolemia | 8,964 (48.3) | 355 (48.0) | 8,609 (48.3) | | | 0.908 |
| Myocardial infarction | 771 (4.2) | 42 (5.7) | 729 (4.1) | | | 0.033 |
| Heart failure | 2,659 (14.3) | 192 (26.0) | 2,467 (13.8) | | | <0.001 |
| Atrial fibrillation | 1,629 (8.8) | 130 (17.6) | 1,499 (8.4) | | | <0.001 |
| ***Medications*** | | | | | | |
| RAS blocker | 9,662 (52.0) | 461 (62,4) | 9,201 (51.6) | | | 0.001 |
| CCB | 4,973 (26.8) | 242 (32.8) | 4,731 (26.5) | | | <0.001 |
| BB | 8,950 (48.2) | 468 (63.3) | 8,482 (47.5) | | | <0.001 |
| Anti-platelet agent | 8,667 (46.7) | 429 (58.1) | 8,238 (46.2) | | | <0.001 |
| Anti-coagulant | 791 (4.3) | 71 (9.6) | 720 (4.0) | | | <0.001 |
| Statin | 7,902 (42.53) | 319 (43.2) | 7,583 (42.5) | | | 0.721 |
| ***Laboratory findings*** | | | | | | |
| Hb, g/dL | 14.2 ± 1.6 | 13.4 ± 1.6 | 14.3 ± 1.6 | | | <0.001 |
| Total cholesterol, mg/dL | 190.3 ± 39.5 | 187.9 ± 40.8 | 190.4 ± 39.5 | | | 0.101 |
| HDL-cholesterol, mg/dL | 51.6 ± 15.9 | 51.5 ± 22.1 | 51.6 ± 15.6 | | | 0.929 |
| LDL-cholesterol, mg/dL | 111.7 ± 43.4 | 109.7 ± 37.0 | 111.8 ± 43.6 | | | 0.188 |
| Triglycerides, mg/dL | 121.9  (121.0 - 122.8) | 121.9  (117.7 - 126.3) | 121.9  (121.0 - 122.8) | | | 0.987 |
| Glucose, mg/dL | 104.2 ± 25.8 | 105.4 ± 40.1 | 104.14 ± 25.1 | | | <0.001 |
| eGFR, mL/min/1.73m^2^ | 81.0 ± 34.1 | 73.44 ± 22.72 | 81.28 ± 34.49 | | | <0.001 |

Values are mean ± standard deviation, median (interquartile range), or n (%). Abbreviations as Supplementary Table 1.
