# Supplementary material for: Augmented risk of dementia in hypertrophic cardiomyopathy: A propensity score matching analysis using the nationwide cohort
Source: PLoS One. 2022 Jun 16;17(6):e0269911. doi: 10.1371/journal.pone.0269911 (PMC9202937; doi:10.1371/journal.pone.0269911)
Supplement: S3 Table — (DOCX) [file pone.0269911.s003.docx]

**Supplementary Table 3.** **The comparison of the impact on the dementia and its subtypes after excluding other cardiac diseases^*^**

|  | **Any type of dementia** | | | **Alzheimer’s disease** | | **Vascular dementia** | |
| --- | --- | --- | --- | --- | --- | --- | --- |
| **Variables** | | **HR (95% CI)** | ***p*** | **HR (95% CI)** | ***p*** | **HR (95% CI)** | ***p*** |
| HCM | | 1.51 (1.07 – 2.15) | 0.021 | 1.59 (1.08 – 2.36) | 0.019 | 1.40 (0.49 – 3.97) | 0.528 |
| Age (per 10 years increment) | | 4.60 (3.78 – 5.60) | <0.001 | 5.05 (4.05 – 6.31) | <0.001 | 3.34 (1.93 – 5.79) | <0.001 |
| Male sex | | 0.36 (0.26 – 0.50) | <0.001 | 036 (0.25 – 0.52) | <0.001 | 0.52 (0.29 – 1.34) | 0.174 |
| Smoking | | 1.58 (1.32 – 1.71) | <0.001 | 1.62 (1.43 – 1.75) | <0.001 | 1.08 (0.36 – 2.39) | 0.867 |
| Heavy drinking | | 1.42 (0.73 – 2.79) | 0.305 | 1.27 (0.62 – 2.60) | 0.518 | 1.49 (0.20 – 11.20) | 0.701 |
| Obesity | | 0.84 (0.60 – 1.17) | 0.301 | 0.88 (0.61 – 1.27) | 0.500 | 0.66 (0.24 – 1.77) | 0.405 |
| Income lower 20% | | 0.90 (0.56 – 1.44) | 0.661 | 0.84 (0.39 – 1.45) | 0.529 | 1.76 (0.58 – 5.41) | 0.321 |
| Hypertension | | 1.70 (1.22 – 2.37) | 0.002 | 1.68 (1.15 – 2.44) | 0.007 | 1.90 (0.70 – 5.14) | 0.206 |
| Diabetes mellitus | | 1.26 (0.80 – 2.01) | 0.324 | 1.38 (0.83 – 2.28) | 0.214 | 1.00 (0.23 – 4.39) | 0.997 |
| Hypercholesterolemia | | 0.86 (0.59 – 1.24) | 0.411 | 0.89 (0.60 – 1.34) | 0.588 | 0.50 (0.14 – 1.75) | 0.278 |
| Prior use of statin | | 0.89 (0.60 – 1.34) | 0.586 | 0.84 (0.53 – 1.34) | 0.465 | 0.77 (0.22 – 2.69) | 0.685 |

^*^Other cardiac diseases include myocardial infarction, heart failure, and atrial fibrillation. CI, confidence interval; HCM, hypertrophic cardiomyopathy; HR, hazard ratio.
